# Discovery of plasma metabolomic biomarkers and a predictive model for immune checkpoint inhibitor-associated myocarditis: a pilot case-control study

**DOI:** 10.3389/fimmu.2026.1915039

**Published:** 2026-09-15

**Authors:** Zhuoling Zheng, Qingling Gu, Yiting Wang, Lihong Huang, Jingwen Xie, Chunmei Dai, Jiali Li, Xiaoyan Li

**Affiliations:** 1 Department of Pharmacy, The Sixth Affiliated Hospital, Sun Yat-Sen University, Guangzhou, Guangdong, China; 2 Guangzhou (Huangpu) Zhongliu Biomedical Innovation Center, Guangzhou, Guangdong, China; 3 School of Pharmaceutical Sciences, Sun Yat-sen University, Guangzhou, Guangdong, China

**Keywords:** biomarkers, gut microbiota, immune checkpoint inhibitors, metabolomics, myocarditis, oxidative stress

## Abstract

**Background:**

Immune checkpoint inhibitor (ICI)-associated myocarditis is a rare but potentially fatal immune-related adverse event lacking reliable biomarkers for risk stratification. This exploratory pilot case-control study aimed to characterize baseline plasma metabolomic profiles in patients who developed ICI-associated myocarditis and to evaluate a metabolite-based predictive model.

**Methods:**

Baseline plasma samples from 15 patients with ICI-associated myocarditis and 15 matched controls were analyzed by untargeted metabolomics using UHPLC-Q/Orbitrap HRMS. Differential metabolites were identified by OPLS-DA and univariate analysis. A metabolite-derived PC1 was used in logistic regression models (clinical-only, metabolite-only, combined), with performance assessed by AUC.

**Results:**

Ten differential metabolites were identified, implicating glutathione metabolism, phenylalanine metabolism, and histidine metabolism pathways. Key alterations included elevated phenylpyruvic acid, 1-methylpseudouridine, cinnamoylglycine, pyroglutamic acid and spermine, with decreased ceramide, cyclo-prolylglycine, propionic acid, glycolic acid, and histamine. A clinical model incorporating three baseline laboratory variables (lymphocyte ratio, monocyte ratio, and eosinophil ratio) achieved an AUC of 0.618. The metabolite PC1 model alone attained an AUC of 0.964, outperforming the clinical model. The combined clinical-metabolite model reached a fitted AUC of 0.973 (cross-validated AUC 0.925). Decision curve analysis indicated that the combined model provided potential net benefit within the 20%-40% threshold range, marginally exceeding the metabolite-only model.

**Conclusion:**

This exploratory study identified baseline plasma metabolites potentially associated with ICI-associated myocarditis risk, implicating oxidative stress, immune modulation, and gut microbiota-host interactions. The metabolite-based model showed preliminary discriminative ability, but prospective validation in independent cohorts is required.

## 1 Introduction

Immune checkpoint inhibitors (ICIs) have become established therapy for various malignancies (1). The immune activation induced by these agents, however, can result in immune-related adverse events (irAEs) affecting multiple organ systems (2, 3). ICI-associated myocarditis, although uncommon, is associated with mortality rates ranging from 25% to 50% and may present hyper-acutely within the first two treatment cycles (4, 5). Clinical manifestations are often nonspecific and may progress rapidly to severe cardiac dysfunction (6, 7). Current diagnosis relies on clinical assessment, cardiac biomarker elevation, electrocardiographic changes, and imaging findings, but these modalities may lack sensitivity for early-stage disease, potentially delaying intervention (8, 9). The identification of biomarkers for risk stratification remains an area of unmet need.

Metabolomics enables systematic characterization of small-molecule metabolites in biological specimens, providing information on metabolic states under pathological conditions (10, 11). This approach has been applied to identify metabolic alterations in cardiovascular diseases, with reported changes involving energy metabolism, lipid profiles, and amino acid pathways (12). Metabolomics studies have begun to explore metabolic alterations in irAEs. Möhn et al. (13) conducted a prospective longitudinal metabolomic analysis and found that baseline levels of specific triglyceride species were significantly lower in patients who subsequently developed irAEs compared with matched controls, particularly in females, suggesting a potential association between lipid metabolism and irAEs. Similarly, Chen et al. (14) reported that elevated baseline acylcarnitines and steroid metabolites predicted irAEs occurrence in patients with lung cancer receiving PD-1/PD-L1 inhibitors, with a prediction model achieving an area under the curve of 0.91. Regarding ICI-associated myocarditis specifically, He et al. (15) revealed increased serum levels of indoleamine 2, 3-dioxygenase-1-derived L-kynurenine in affected patients compared with controls through untargeted metabolomic profiling. Although these studies provide preliminary evidence, the plasma metabolomic profile of ICI-associated myocarditis remains incompletely characterized, and predictive models integrating metabolite scores with clinical variables are lacking.

In this study, untargeted metabolomic profiling was performed using baseline plasma samples from 15 patients with ICI-associated myocarditis and 15 matched controls from an institutional cohort (16). Ultra-high-performance liquid chromatography coupled with quadrupole/electrostatic field orbitrap high-resolution mass spectrometry (UHPLC-Q-Exactive Orbitrap MS) was employed. Given the small sample size, this analysis was designed as exploratory. The objectives were: (1) to compare plasma metabolic profiles between groups and identify differentially abundant metabolites; (2) to evaluate the discriminatory performance of a metabolite score; and (3) to assess whether metabolomic data could provide additional information beyond clinical variables.

## 2 Material and methods

### 2.1 Study population and samples

This study was based on a previously published retrospective case-control study by our team (16). That study enrolled patients with malignant tumors who received ICI therapy at The Sixth Affiliated Hospital, Sun Yat-sen University, Guangzhou, China between January 2020 and December 2024, and identified 98 patients with ICI-associated myocarditis and 98 controls without myocarditis. The parent study was unmatched. For the present metabolomics study, we selected 15 myocarditis patients and 15 controls from the biobank of the above cohort based on the availability of baseline plasma samples, and then performed 1:1 matching on age, sex, cancer type, and ICI regimen to minimize potential confounding. All participants provided written informed consent for biospecimen collection. Baseline plasma samples were collected within 24 hours prior to the first ICI infusion. Peripheral venous blood (5 mL) was drawn into EDTA-containing tubes, centrifuged at 4000 rpm for 10 min at 4 °C, and the plasma was separated and stored at -80 °C until analysis. Inclusion criteria were consistent with our previous study: [1] availability of complete clinical data; and [2] receipt of at least one cycle of ICI therapy. Exclusion criteria were: [1] incomplete clinical data; [2] abnormal cardiac injury markers or electrocardiogram findings attributable to other diseases; or [3] pre-existing severe organ dysfunction prior to ICI initiation. This was a retrospective case-control study, and the patient selection process complied with the STROBE statement. The study protocol was approved by the Ethics Committee of The Sixth Affiliated Hospital, Sun Yat-sen University, Guangzhou, China (Approval No. 2024ZSLYEC-524).

### 2.2 Data collection and myocarditis definitions

Clinical data were retrospectively extracted from the electronic medical record system, covering the following domains: [1] demographic characteristics and medical history; [2] cancer treatment-related information, including cancer type, ICI agent, combination regimen, and line of therapy; [3] and cardiac-related parameters, such as cardiac enzyme profiles and electrocardiogram (ECG). All data were collected at baseline, prior to the first ICI infusion. The diagnosis of myocarditis was made according to the 2025 European Society of Cardiology Guidelines (17). Definite myocarditis required a compatible clinical presentation confirmed by cardiac magnetic resonance (CMR) or endomyocardial biopsy (EMB); possible myocarditis was defined as a compatible clinical presentation with at least one additional diagnostic criterion (e.g., ECG abnormalities, elevated cardiac troponin, imaging abnormalities) when CMR or EMB were unavailable or inconclusive. Patients with definite or possible ICI-associated myocarditis were included in the case group, while those with an unlikely diagnosis were excluded.

### 2.3 Plasma metabolites detection

An aliquot of 50 μL plasma was mixed with 200 μL pre-cooled acetonitrile, vortexed for 1 min, and kept at 4 °C for 5 min. After centrifugation at 15, 000 rpm and 4 °C for 10 min, 200 μL of the supernatant was collected and dried under vacuum. The dried residue was stored at -80 °C and analyzed within one month. Equal volumes of all plasma samples were pooled and processed in parallel as quality control (QC) samples. A QC sample was injected before and after every eight test samples, and 24 samples constituted one analytical batch. On the day of analysis, the dried residues were reconstituted in 100 μL of methanol/water (1:9), vortexed for 1 min, placed at 4 °C for 5 min, and then centrifuged at 15, 000 rpm and 4 °C for 10 min. The supernatant was transferred to autosampler vials and kept at 8 °C until LC-MS analysis.

Metabolic profiling was performed using an Ultimate 3000 UHPLC system coupled with a Q-Exactive Orbitrap mass spectrometer (Thermo Scientific). Chromatographic separation was carried out on an ACQUITY UPLC HSS T3 column (100 mm × 2.1 mm, 1.8 μm, Waters). For positive ion mode, mobile phase A was water containing 0.1% formic acid and mobile phase D was acetonitrile containing 0.1% formic acid. The gradient elution was: 0–1 min, 5% D; 1–22 min, linear to 100% D; 22–26 min, 100% D; 26-26.1 min, back to 5% D; 26.1–30 min, 5% D. For negative ion mode, mobile phase B was water/methanol (5:95, v/v) and mobile phase C was water with 6.5 mM ammonium formate. The gradient was: 0–1 min, 2% B; 1–18 min, linear to 100% B; 18–22 min, 100% B; 22-22.1 min, back to 2% B; 22.1–25 min, 2% B. The column temperature was set at 50 °C for positive mode and 55 °C for negative mode. The injection volume was 5 μL and the flow rate was 0.35 mL/min for both modes. The needle was washed with 200 μL of 50% methanol/water three times before and after each injection. Of note, the ACQUITY UPLC HSS T3 column used in this study is a reversed-phase non-chiral column and does not resolve enantiomeric isomers.

Mass spectrometry parameters were as follows: capillary temperature 325 °C, auxiliary gas temperature 350 °C, scan range m/z 70–1050. The full-scan resolution was 70, 000 and the data-dependent MS/MS (ddMS2) resolution was 17, 500. The top 10 most intense ions from each full scan were selected for fragmentation using stepped normalized collision energies of 15, 30, and 45. The instrument was tuned prior to use.

Raw data were processed using MS-DIAL software. Metabolite identification was based on the ESI (±)-MS/MS authentic standards library (version 17) provided by MS-DIAL. Peak areas were corrected by the Systematic Error Removal using Random Forest (SERRF) method before subsequent statistical analysis.

### 2.4 Statistical analysis

#### 2.4.1 Data preprocessing

Raw metabolomics data processed with MS-DIAL were filtered to remove redundant adducts within an m/z window of 0.0001 and to exclude metabolites detected in < 30% of participants. After SERRF correction, data were log_2_(x+1) transformed and Pareto scaled. For metabolites with zero or missing peak areas in some samples, the value 0 was directly entered into the log_2_(x+1) transformation without additional imputation. To visualize overall metabolic differences between groups prior to supervised modeling, unsupervised principal component analysis (PCA) was performed on all filtered features in each ion mode, with samples colored by group and 95% confidence ellipses added.

#### 2.4.2 Discovery of differential metabolites

Orthogonal partial least-squares discriminant analysis (OPLS-DA) was used to assess metabolic differences between groups, with performance evaluated by seven-fold cross-validation (R^2^Y, Q^2^). Variable importance in projection (VIP) scores quantified metabolite contributions (VIP > 1 considered important). Univariate differential analysis was performed with the limma empirical Bayes model to estimate log_2_-fold changes (logFC) and Benjamini-Hochberg false discovery rate (FDR)-adjusted p values (*q* value). Differential metabolites were defined as |logFC| > 1 and *q*-value < 0.05. PCA of these metabolites extracted the first principal component (PC1) as a comprehensive metabolic index.

#### 2.4.3 Pathway enrichment analysis

PubChem IDs of differential metabolites were submitted to MetaboAnalyst (https://www.metaboanalyst.ca). Species was set to Homo sapiens, and enrichment was performed against the KEGG human metabolic pathway library using the hypergeometric test with Benjamini-Hochberg FDR correction (FDR < 0.05 significant). Results were visualized as a bubble plot. Metabolites not mapped to KEGG were excluded from enrichment but remained in the differential metabolite list.

#### 2.4.4 Clinical-metabolite combined prediction model

Based on our previous findings (16), three hematological ratios (lymphocyte ratio, monocyte ratio, and eosinophil ratio) were dichotomized (abnormal *vs*. normal). All three hematological ratios were calculated as the percentage of each cell type relative to total leukocytes. Myoglobin was also considered but was excluded because all patients in the present cohort had normal baseline levels, preventing dichotomization. Normal reference ranges were defined as follows: lymphocyte ratio 20%-40%, monocyte ratio 3%-10%, eosinophil ratio 0.5%-5%, and myoglobin ≤ 106 ng/mL. Values outside these ranges were classified as abnormal. Three logistic regression models were built: clinical-only, metabolite-PC1-only, and combined. Ten-fold cross-validation (5 repeats) evaluated discriminative ability; mean area under the receiver operating characteristic curve (AUC) with 95% confidence interval (CI) was calculated. ROC curves compared model performance. Goodness-of-fit was assessed by the Hosmer-Lemeshow test. Bootstrap resampling (1000 replicates) derived 95% CIs for AUC, sensitivity, and specificity of the combined model. LASSO regression was explored as a sensitivity analysis but yielded an unstable regularization path given the limited sample size; standard logistic regression results are therefore reported with explicit acknowledgment of overfitting risk.

#### 2.4.5 Model evaluation and visualization

Calibration curves with LOESS smoothing assessed agreement between predicted probabilities and observed outcomes of the combined model. Decision curve analysis (DCA) calculated net benefits across threshold probabilities, comparing the combined model, clinical-only model, “treat-all” and “treat-none” strategies. A nomogram based on the combined model was constructed using the rms package to visualize predictor contributions and calculate predicted risk of ICI-associated myocarditis.

#### 2.4.6 Software and statistical environment

All statistical analyses were performed using R software (version 4.6.0). The following R packages were employed: ropls, limma, pROC, caret, dcurves, and rms. A two-sided *p* value < 0.05 was considered statistically significant. The Benjamini-Hochberg procedure was applied to control the FDR for multiple comparisons.

## 3 Results

### 3.1 Patient characteristics

This study selected 15 patients with ICI-associated myocarditis and 15 controls with available baseline plasma samples from our previously published clinical cohort. All patients received ICI therapy at The Sixth Affiliated Hospital, Sun Yat-sen University, Guangzhou, China between January 2020 and December 2024. Baseline clinical characteristics of the two groups are summarized in **Table 1**. No statistically significant differences were observed between the myocarditis and control groups in age, sex, body mass index (BMI), cancer type, tumor-node-metastasis (TNM) stage, Eastern Cooperative Oncology Group (ECOG) performance status, ICI agent, line of therapy, combination regimens, baseline electrocardiogram (ECG) status, or cardiovascular risk factors. The mean age was 59.07 ± 7.03 years in the myocarditis group and 55.20 ± 4.06 years in the control group; male patients accounted for 46.67% and 66.67%, respectively. The mean BMI was similar between groups (22.82 ± 3.35 *vs.* 21.69 ± 3.68, *p* = 0.387). The most common cancer types were colon cancer, rectal cancer and gastric cancer. Advanced stage (III-IV) disease was observed in 60.00% of myocarditis patients and 86.67% of controls (*p* = 0.215). ECOG performance status was balanced between the two groups (OR = 3.50, 95% CI: 0.32-38.23, *p* = 0.598). ECOG 1 was observed in 80.00% of myocarditis patients and 93.33% of controls; ECOG 2 was observed in the remaining patients. Sintilimab was the most frequently administered ICI (60.00% in the myocarditis group and 73.33% in the control group). Baseline ECG abnormalities were present in 7 (46.67%) patients in the myocarditis group and 3 (20.00%) in the control group, with no significant difference between groups (OR = 3.50, 95% CI: 0.69-17.71, *p* = 0.121). All patients in both groups had baseline myoglobin levels within the normal reference range.

**Table 1:** Baseline demographic and clinical characteristics of the study population (n = 30).

Variables	Total (n = 30)	Myocarditis (n = 15)	Control (n = 15)	OR (95% CI)	*P* value
Demographic characteristics					
Age at start of ICI (years), mean ± SD	57.13 ± 5.97	59.07 ± 7.03	55.20 ± 4.06	–	0.075^†^
Male, *n* (%)	17 (56.67)	7 (46.67)	10 (66.67)	0.44 (0.10-1.92)	0.269^ǂ^
BMI (kg/m^2^), mean ± SD	22.26 ± 3.50	22.82 ± 3.35	21.69 ± 3.68	–	0.387^†^
Types of cancer, n (%)					
Colon cancer	14 (46.67)	7 (46.67)	7 (46.67)	0.99 (0.24-4.20)	0.999^ǂ^
Rectal cancer	10 (33.33)	4 (26.67)	6 (40.00)	0.55 (0.12-2.55)	0.439^ǂ^
Gastric cancer	5 (16.67)	3 (20.00)	2(13.33)	1.63 (0.23-14.46)	0.999^§^
Esophageal cancer	1 (3.33)	1 (6.67)	0 (0)	–	0.999^§^
TNM stage (III/IV), n (%)	22 (73.33)	9 (60.00)	13 (86.67)	0.23 (0.04-1.41)	0.215^§^
ECOG performance score, n (%)					
1	26 (86.67)	12 (80.00)	14 (93.33)	ref	–
2	4 (13.33)	3 (20.00)	1 (6.67)	3.50 (0.32-38.23)	0.598^§^
≥ 3	0	0	0	–	–
Treatment lines of ICI, *n* (%)					
Neoadjuvant therapy	5 (16.67)	4 (26.67)	1 (6.67)	5.09 (0.50-52.29)	0.330^§^
Adjuvant therapy	3 (10.00)	2 (13.33)	1 (6.67)	2.15 (0.17-26.67)	0.999^§^
First-line therapy	8 (26.67)	4 (26.67)	4 (26.67)	0.99 (0.20-5.01)	0.999^§^
Second-line therapy	7 (23.33)	1 (6.67)	6 (40.00)	0.11 (0.01-1.04)	0.080^§^
≥Third-line therapy	7 (23.33)	4 (26.67)	3 (20.00)	1.46 (0.26-8.01)	0.999^§^
Type of ICIs, *n* (%)					
Sintilimab	20 (66.67)	9 (60.00)	11 (73.33)	0.55 (0.12-2.55)	0.439^ǂ^
Toripalimab	3 (10.00)	3 (20.00)	0 (0)	–	0.224^§^
Tislelizumab	2 (6.67)	2 (13.33)	0 (0)	–	0.483^§^
Camrelizumab	2 (6.67)	1 (6.67)	1(6.67)	0.99 (0.06-17.62)	0.999^§^
Others	3 (10.00)	0 (0)	3 (20.00)	–	0.224^§^
Combined therapy, n (%)					
ICI monotherapy	6 (20.00)	3 (20.00)	3 (20.00)	0.99 (0.17-5.99)	0.999^§^
Chemotherapy	13 (43.33)	7 (46.67)	6 (40.00)	1.31 (0.31-5.58)	0.713^ǂ^
Targeted-therapy	4 (13.33)	2 (13.33)	2 (13.33)	0.99 (0.12-8.21)	0.999^§^
Chemotherapy plus targeted therapy	7 (23.33)	3 (20.00)	4 (26.67)	0.69 (0.13-3.79)	0.999^§^
Baseline ECG status, n (%)					
Abnormal ECG	10 (33.33)	7 (46.67)	3 (20.00)	3.50 (0.69-17.71)	0.121^ǂ^
Cardiovascular risk factors, *n* (%)					
Hypertension	6 (20.00)	3 (20.00)	3 (20.00)	0.99 (0.17-5.99)	0.999^§^
Diabetes	3 (10.00)	1 (6.67)	2 (13.33)	0.46 (0.04-5.75)	0.999^§^
Prior CV diseases	4 (13.33)	4 (26.67)	0 (0)	–	0.100^§^

Data are presented as mean ± SD for continuous variables and n (%) for categorical variables.

†Student’s t-test; ǂ Pearson Chi-square test; § Fisher’s exact test. For categorical variables, Pearson Chi-square test was used when all expected cell counts ≥ 5; Fisher’s exact test was applied when expected cell count < 5. ORs and 95% CIs were calculated using logistic regression.

BMI, body mass index; CI, confidence interval; CV, cardiovascular; ECG, electrocardiogram; ECOG, Eastern Cooperative Oncology Group; ICI, immune checkpoint inhibitor; OR, odds ratio; SD, standard deviation; TNM, tumor-node-metastasis.

### 3.2 Metabolomics data quality and screening of differential metabolites

To assess data quality, a QC sample was injected after every eight analytical runs. In positive ion mode, the median relative standard deviation (RSD) of peak areas among QC samples was 19.0%. After removing metabolites with RSD > 30%, 242 metabolites were retained, and the median RSD decreased to 11.6%. In negative ion mode, the median QC RSD was 20.4%; following the same filtering criterion, 148 metabolites were retained, and the median RSD dropped to 9.2%. PCA of all samples showed that QC samples clustered relatively tightly in both ion modes (**Supplementary Figure 1**), indicating that the filtered data were of good quality and suitable for subsequent analyses.

Unsupervised PCA of all filtered features in each ion mode, with samples colored by group, showed moderate separation between the myocarditis and control groups (**Supplementary Figure 2**), suggesting that baseline metabolic profiles differed between the two groups at the global level.

To evaluate the metabolic profile differences between the myocarditis and control groups, an OPLS-DA model was constructed. The OPLS-DA score plots demonstrated separation between the two groups in both positive and negative ion modes (**Figure 1**). In positive ion mode, the model yielded an R^2^Y of 0.900 and a Q^2^ of 0.621; in negative ion mode, the R^2^Y was 0.795 and the Q^2^ was 0.435. The Q^2^ value in negative ion mode fell below the conventional threshold of 0.50, suggesting that the predictive robustness of this model is relatively limited. Nevertheless, permutation tests (200 permutations) showed that the original Q^2^ values of both models were higher than those of all permuted models (*p* = 0.005, **Supplementary Figure 3**), indicating that the observed separation, while modest in predictive strength, was unlikely to arise from random noise.

**Figure 1:**
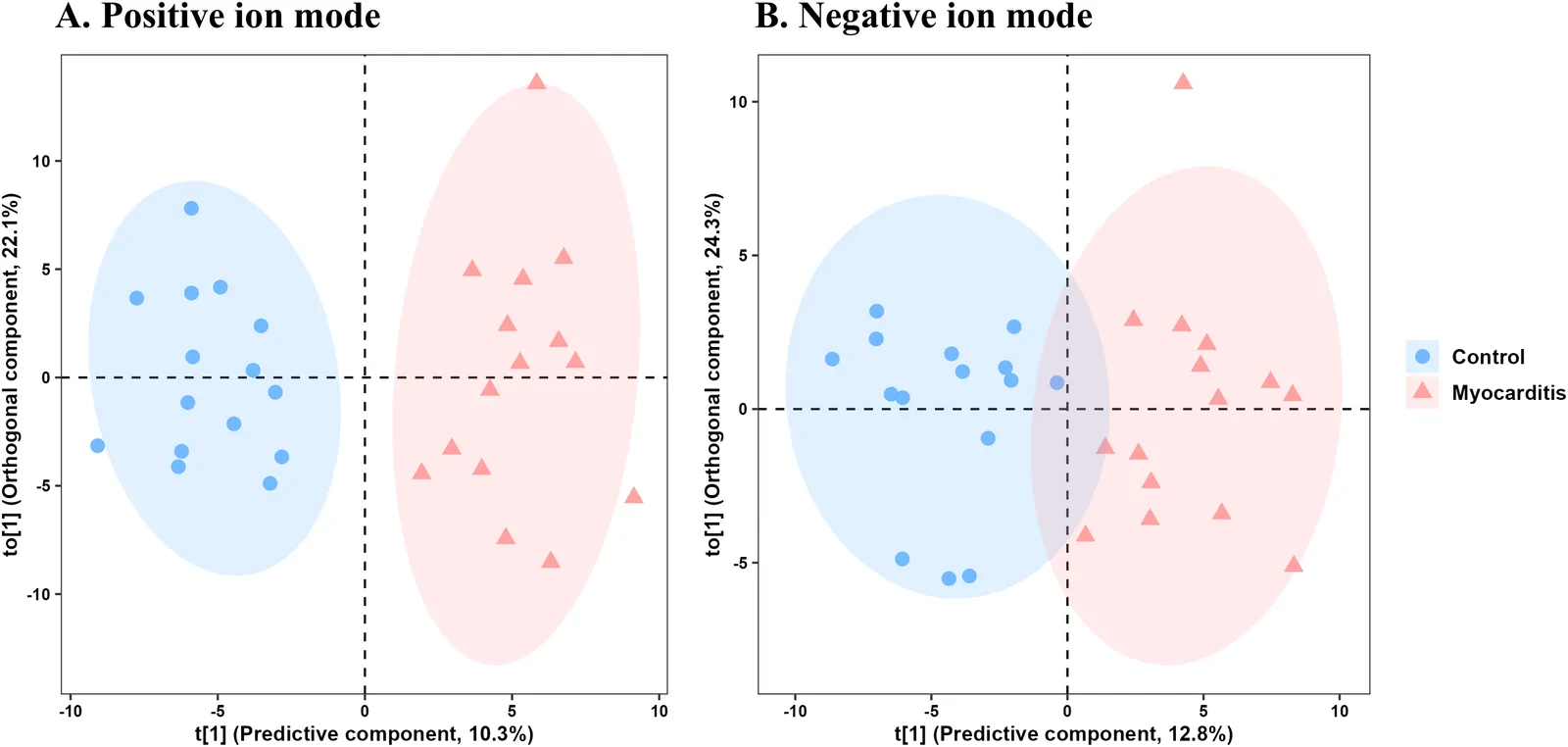
Orthogonal partial least squares discriminant analysis (OPLS-DA) score plots of metabolomic profiles in positive **(A)** and negative **(B)** ion modes. Samples were preprocessed by log_2_ transformation and Pareto scaling. **(A)** Positive ion mode with t[1] (predictive component, 10.3%) and to[1] (orthogonal component, 22.1%). **(B)** Negative ion mode with t[1] (predictive component, 12.8%) and to[1] (orthogonal component, 24.3%). Blue circles, control group; red triangles, myocarditis group. Model performance was evaluated by cross-validation (positive mode: R²Y = 0.900, Q² = 0.621; negative mode: R²Y = 0.795, Q² = 0.435).

To more comprehensively illustrate the metabolites contributing to model discrimination, heatmaps were generated using the top 30 metabolites ranked by variable importance in projection (VIP) scores in each ion mode (**Figure 2**). Cluster analysis revealed that these high-VIP metabolites distinguished the myocarditis group from the control group to a certain extent, with the two groups largely forming separate clusters. The relative abundance patterns of these metabolites within each group showed high consistency, further supporting the discriminative capacity of the OPLS-DA models.

**Figure 2:**
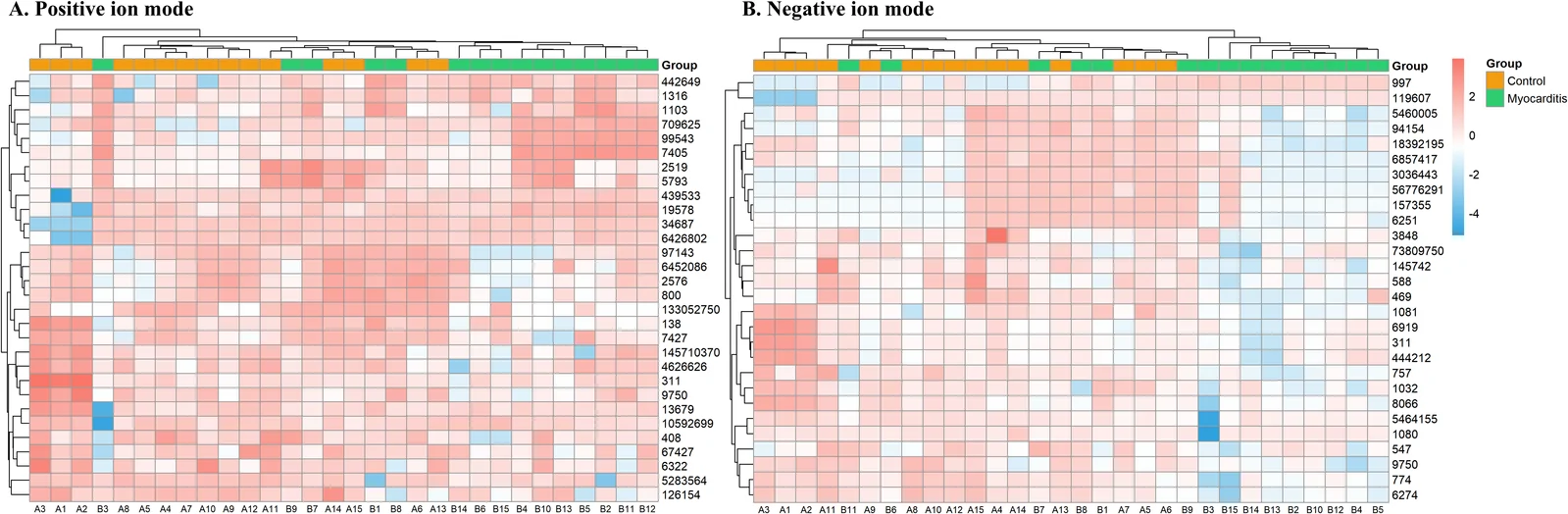
Heatmaps of the top 30 metabolites ranked by variable importance in projection (VIP) scores in positive **(A)** and negative **(B)** ion modes. Rows represent individual metabolites (PubChem IDs) and columns represent individual samples. Sample IDs are shown on the x-axis: A1-A15 indicate control samples; B1-B15 indicate myocarditis samples. The color scale indicates relative metabolite abundance after log_2_ transformation and Pareto scaling. Red, high abundance; blue, low abundance.

Using the criteria of VIP > 1, |log_2_FC| > 1, and *q* < 0.05 in the OPLS-DA model, a total of ten differential metabolites were identified (**Table 2**). The volcano plot illustrates the distribution of these metabolites between the two groups (**Figure 3**), among which five were upregulated and five were downregulated. These metabolites encompass various classes, including amino acid metabolism (phenylpyruvic acid, pyroglutamic acid, cyclo-prolylglycine), lipid metabolism (ceramide), nucleotide metabolism (1-methylpseudouridine), organic acid metabolism (propionic acid, glycolic acid), bioactive amine metabolism (histamine), polyamine metabolism (spermine), and gut microbiota-host co-metabolism (cinnamoylglycine). To intuitively illustrate the relative abundance distribution of each differential metabolite between the two groups, violin plots were generated (**Figure 4**). Wilcoxon rank-sum test results showed that the relative abundance of all differential metabolites differed significantly between the myocarditis and control groups (*p* < 0.05). Among them, the relative abundance of phenylpyruvic acid, 1-methylpseudouridine, cinnamoylglycine, pyroglutamic acid, and spermine were significantly higher in the myocarditis group, whereas those of ceramide, cyclo-prolylglycine, propionic acid, glycolic acid, and histamine were significantly lower in the myocarditis group.

**Table 2:** Differential metabolites identified in baseline plasma between ICI-associated myocarditis patients and controls.

Metabolite	PubChem ID	VIP	Log_2_FC	AveExpr	*t* statistic	*p* value	*q* value	Ion mode
Phenylpyruvic acid	997	5.651	12.135	12.720	4.981	< 0.001	0.003	Negative
1-Methylpseudouridine	99543	4.292	6.216	19.385	4.139	< 0.001	0.005	Positive
Cinnamoylglycine	709625	4.197	7.506	12.269	3.935	< 0.001	0.007	Positive
Pyroglutamic acid^*^	7405	2.653	3.379	26.491	3.802	< 0.001	0.009	Positive
Ceramide	5283564	3.224	-4.463	15.696	-3.075	0.005	0.040	Positive
Cyclo-Prolylglycine	126154	2.114	-2.334	17.241	-3.387	0.002	0.024	Positive
Spermine	1103	1.696	1.569	19.385	4.344	< 0.001	0.003	Positive
Propionic Acid	1032	1.629	-1.620	19.576	-3.762	< 0.001	0.018	Negative
Glycolic Acid	757	1.598	-1.204	19.712	-4.793	< 0.001	0.003	Negative
Histamine	774	1.567	-1.029	19.607	-3.912	< 0.001	0.018	Negative

Differential metabolites were defined as those with VIP > 1, |log_2_FC| > 1, and FDR-adjusted *q* value < 0.05 (Benjamini-Hochberg correction). All metabolites were identified based on MS-DIAL spectral library matching against the ESI (±)-MS/MS authentic standards library (version 17).

*Pyroglutamic acid (PubChem ID 7405) was identified as [M+H]^+^ (m/z 130.05) with authentic MS/MS spectral support. A negative-mode feature at m/z 128.03 (PubChem ID 439685) was also matched to pyroglutamic acid; it is omitted from **Table 2** to avoid duplicate reporting of the same metabolite. Because the non-chiral reversed-phase column cannot resolve enantiomers, the metabolite is reported as pyroglutamic acid without stereochemical designation. Biological interpretation is based on L-pyroglutamic acid as the representative bioactive form.

AveExpr, average log_2_-transformed abundance across all samples; log_2_FC, log_2_ fold change (positive value indicates higher abundance in myocarditis group); VIP, variable importance in projection.

**Figure 3:**
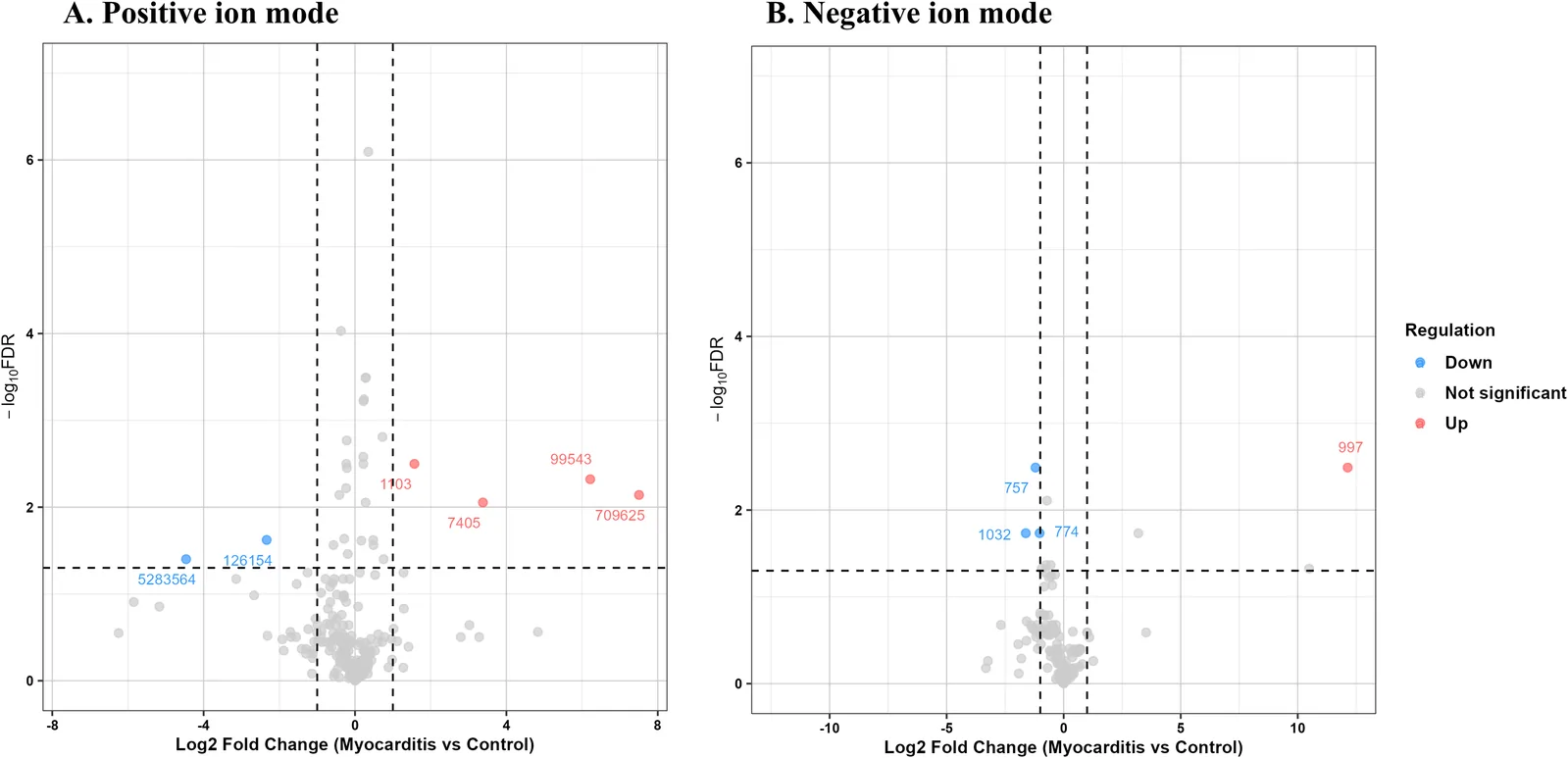
Volcano plots of differential metabolites in positive **(A)** and negative **(B)** ion modes. The x-axis represents log_2_ fold change (myocarditis *vs.* control) and the y-axis represents -log_10_(false discovery rate, FDR). Dashed lines indicate the thresholds for significance (|log_2_FC| > 1, *q* < 0.05). Red dots, significantly upregulated metabolites; blue dots, significantly downregulated metabolites; gray dots, non-significant metabolites. Metabolites with VIP > 1 are labeled with PubChem IDs. See **Table 2** for the correspondence between PubChem IDs and metabolite names.

**Figure 4:**
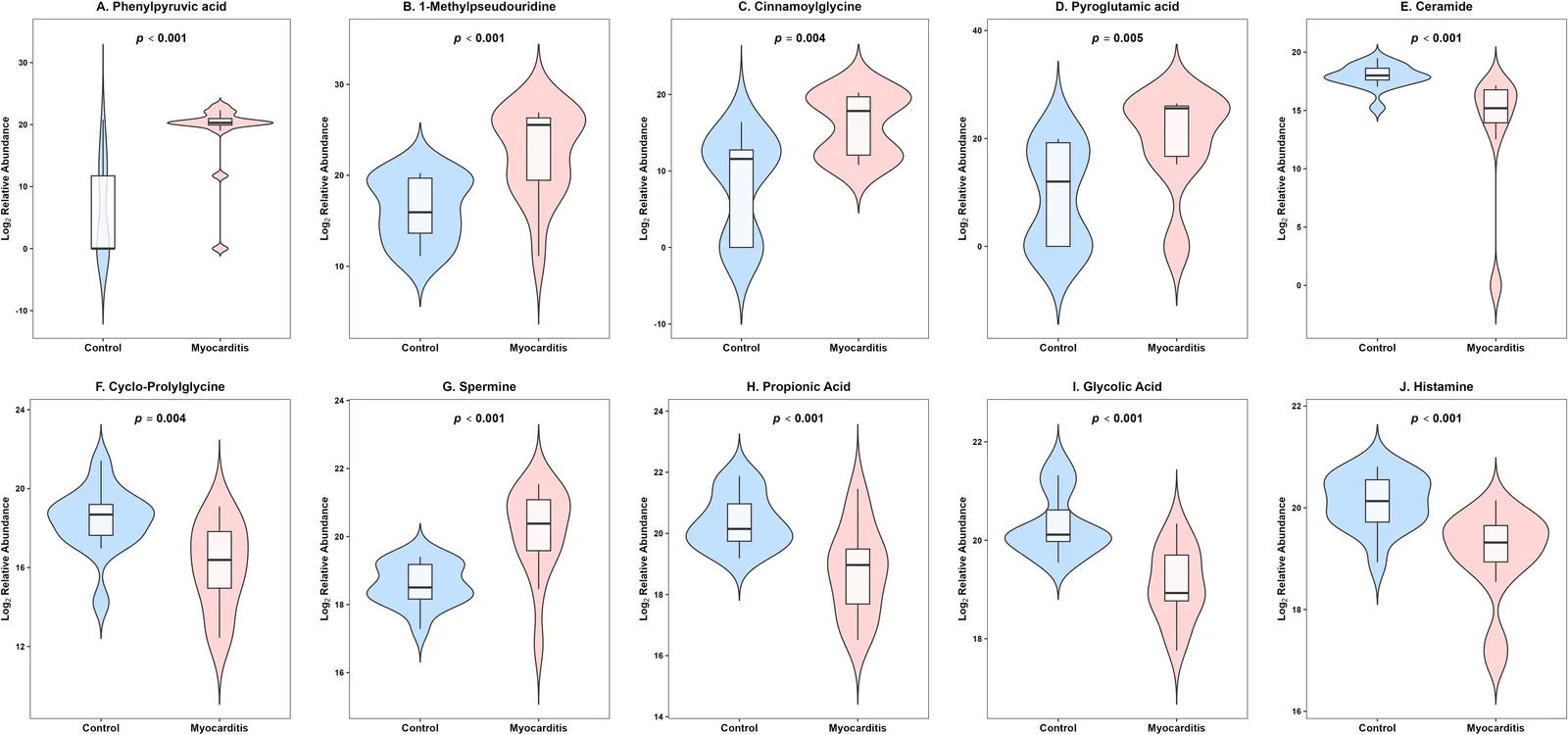
Violin plots showing the relative abundance of 10 differential metabolites between the control (blue) and myocarditis (red) groups. **(A)** phenylpyruvic acid, **(B)** 1-methylpseudouridine, **(C)** cinnamoylglycine, **(D)** pyroglutamic acid, **(E)** ceramide, **(F)** cyclo-prolylglycine, **(G)** spermine, **(H)** propionic acid, **(I)** glycolic acid, and **(J)** histamine. The y-axis represents log_2_-transformed relative abundance. Violin width indicates data density; box plots within each violin indicate the median (center line) and interquartile range (box limits). *P* values were calculated using the Mann-Whitney U test. Biological interpretation of pyroglutamic acid is based on L-pyroglutamic acid as the representative bioactive form.

To provide additional context for interpreting the differential metabolites, detection rates for each metabolite in the myocarditis and control groups are summarized in **Supplementary Table 1**.

PCA was performed separately on the differential metabolites identified in positive and negative ion modes. The PC1 explained 57.2% and 67.7% of the total variance, respectively (**Supplementary Figure 4**). In both modes, the PCA score plots showed separation between the myocarditis and control groups along the PC1 axis, with higher PC1 scores in the myocarditis group (*p* < 0.001). Loading analysis indicated that in positive ion mode, 1-methylpseudouridine, pyroglutamic acid and spermine contributed most to PC1, whereas in negative ion mode, phenylpyruvic acid, glycolic acid, and propionic acid exhibited the highest loadings (**Supplementary Figure 5**).

### 3.3 Pathway enrichment analysis

Given the limited number of differential metabolites (n = 10), this study employed an exploratory KEGG pathway analysis to suggest potential biological directions. KEGG enrichment analysis of the differential metabolites identified seven metabolic pathways, with glutathione metabolism being the most significantly enriched, followed by phenylalanine, tyrosine and tryptophan biosynthesis, phenylalanine metabolism, histidine metabolism, β-alanine metabolism, propanoate metabolism, and arginine and proline metabolism (**Figure 5**). Notably, the inclusion of phenylalanine-related pathways is consistent with phenylpyruvic acid being the top-ranked differential metabolite. Specifically, pyroglutamic acid was mapped to glutathione metabolism, phenylpyruvic acid to phenylalanine metabolism and phenylalanine, tyrosine and tryptophan biosynthesis, propionic acid to propanoate metabolism, histamine to histidine metabolism, and spermine to arginine and proline metabolism.

**Figure 5:**
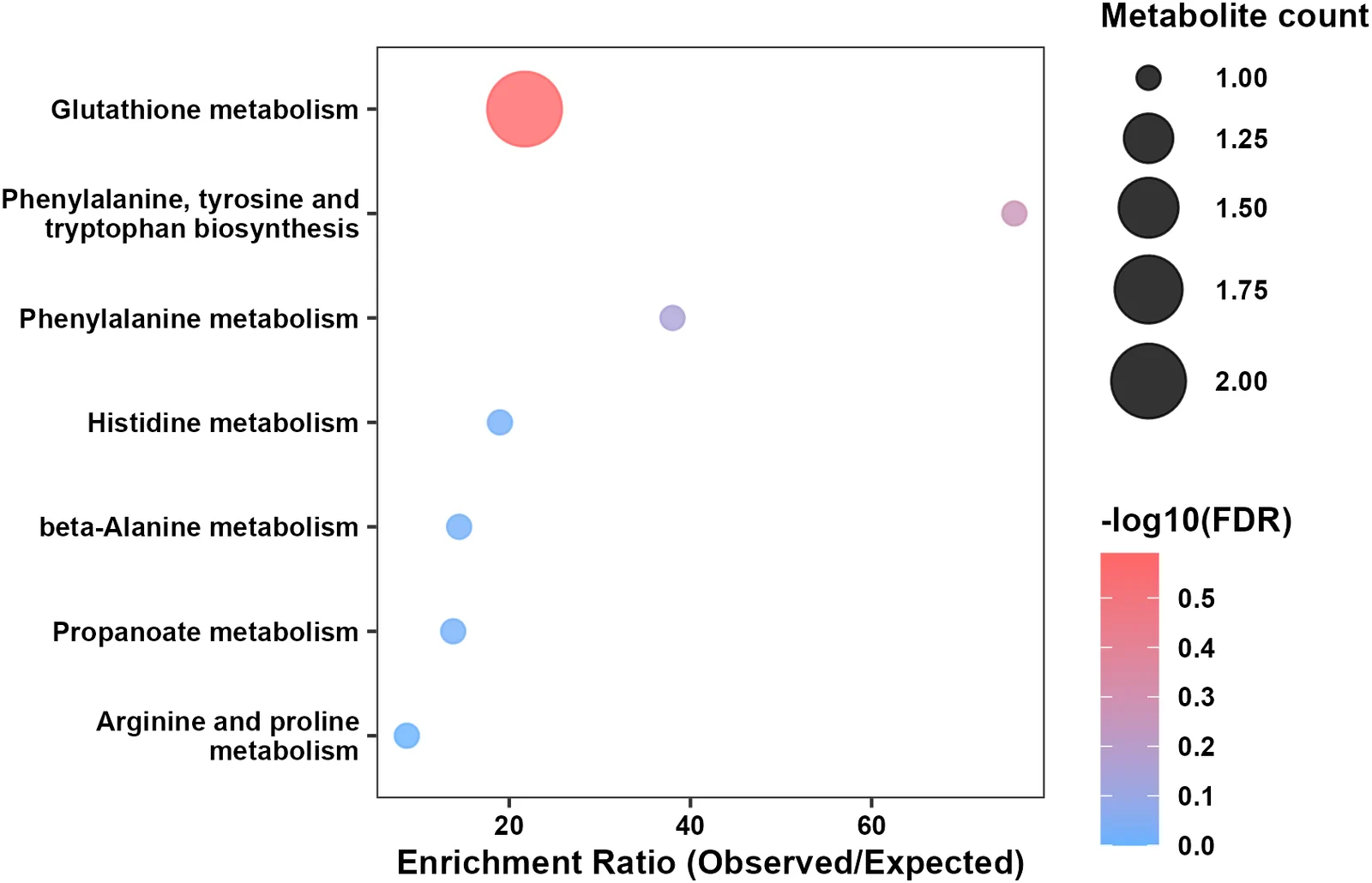
KEGG pathway enrichment analysis of the differential metabolites. The x-axis indicates the enrichment ratio (observed/expected metabolites), the y-axis shows enriched pathways, the dot size represents the number of metabolites mapped to each pathway, and the color scale indicates-log_10_(FDR). Pathways with FDR < 0.05 (Benjamini-Hochberg corrected) were considered significantly enriched. Metabolites not mappable to KEGG pathways were excluded from enrichment analysis.

### 3.4 Combined clinical and metabolite prediction model

To evaluate whether metabolomic data could enhance the prediction of ICI-associated myocarditis, we constructed three logistic regression models: (1) a clinical model incorporating lymphocyte ratio, monocyte ratio, and eosinophil ratio. Myoglobin was not included because all patients had normal baseline values. (2) a metabolite model using metabolite PC1 as the sole predictor; and (3) a joint model integrating both clinical variables and metabolite PC1.

ROC curve analysis revealed that the metabolite model alone demonstrated apparent discriminatory performance (AUC = 0.964, 95% CI: 0.908-1.000, **Figure 6A**), showing higher performance than the clinical model (AUC = 0.618, 95% CI: 0.423-0.813). Critically, this AUC is not unbiased: PC1 was derived from metabolites preselected for group differences, and evaluating it in the same 30 samples largely restates the selection step. The joint model further increased the apparent AUC to 0.973 (95% CI: 0.928-1.000), suggesting that metabolomic information may provide incremental predictive value beyond clinical parameters. A nomogram derived from the joint model (**Figure 6B**) was developed to facilitate individualized risk assessment. Since all patients in this matched cohort had normal baseline myoglobin levels, this variable could not be dichotomized and was therefore excluded from the nomogram. The clinical model in the nomogram was constructed using the three available hematological ratios (lymphocyte ratio, monocyte ratio, and eosinophil ratio).

**Figure 6:**
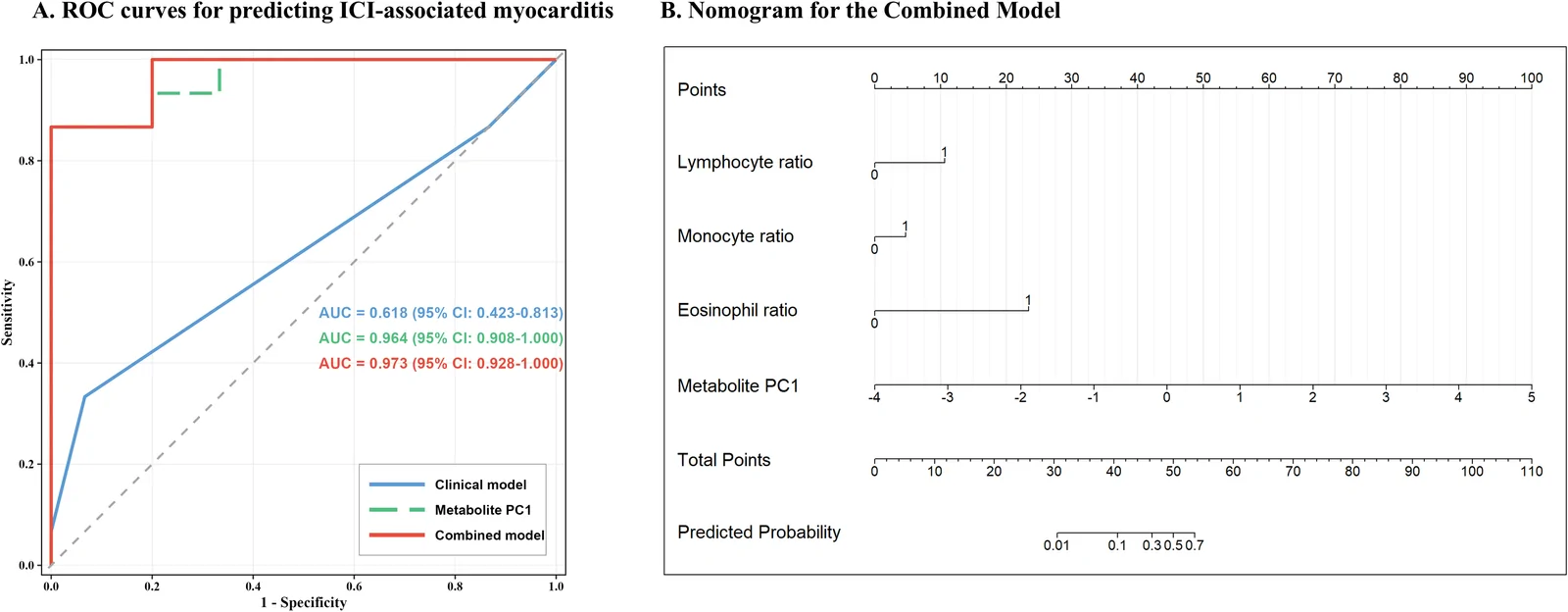
Predictive performance of clinical, metabolite, and combined models for immune checkpoint inhibitor (ICI)-associated myocarditis. **(A)** Receiver operating characteristic (ROC) curves comparing the discriminatory ability of the clinical model (blue), metabolite principal component 1 (PC1) model (green), and combined model (red). The dashed diagonal line represents the reference line (AUC = 0.5). **(B)** Nomogram for individualized risk prediction based on the combined model, incorporating lymphocyte ratio, monocyte ratio, eosinophil ratio, and metabolite PC1. All hematological ratios are expressed as the percentage of total leukocytes, dichotomized as abnormal *vs*. normal based on reference ranges (lymphocyte ratio 20% - 40%, monocyte ratio 3% - 10%, eosinophil ratio 0.5% - 5%). To use the nomogram, locate each variable value on the corresponding axis, draw a vertical line upward to the “Points” axis to determine the score, sum the points for all variables, and locate the total score on the “Total Points” axis to obtain the predicted probability of myocarditis.

The calibration curve of the combined model revealed a broad spread of predicted probabilities (**Supplementary Figure 6**). Given the small sample size, calibration metrics are reported for descriptive purposes only: calibration intercept = 0, calibration slope = 1.001, and Brier score = 0.072. The combined model achieved a fitted AUC of 0.973 and a cross-validated AUC of 0.925; the decrement between fitted and cross-validated estimates suggests potential overfitting. However, this cross-validated (CV) AUC does not fully eliminate the optimistic bias, because feature selection was not embedded within the CV procedure.

DCA (**Supplementary Figure 7**) showed that within the clinical decision threshold range of 20%–40%, the combined model achieved the highest mean net benefit (0.233), followed by the metabolite model (0.231), while the clinical model had the lowest (0.183). The absolute increase in mean net benefit of the combined model compared with the clinical model was 0.050, representing a relative improvement of approximately 27.3%. The net benefit curves of the combined model and the metabolite model were closely aligned within this threshold range, suggesting that metabolite PC1 is a major contributor driving the decision benefit of the model.

## 4 Discussion

This exploratory case-control study performed untargeted metabolomic profiling on baseline plasma samples from 15 patients with ICI-associated myocarditis and 15 controls. Preliminary results suggest that certain differences in plasma metabolic profiles may exist between the two groups, with a total of ten differential metabolites identified, involving pathways such as glutathione metabolism, histidine metabolism, beta-alanine metabolism, propanoate metabolism, as well as arginine and proline metabolism. The first principal component extracted from these metabolites suggested potential discriminative ability in this dataset, and its combination with clinical variables marginally improved model performance. These findings are hypothesis-generating and require validation in larger, prospective cohorts. All results should therefore be interpreted as exploratory and hypothesis-generating, pending orthogonal validation.

Among the differential metabolites, pyroglutamic acid was markedly elevated in the myocarditis group. However, L-pyroglutamic acid is the biologically active enantiomer in humans and a key intermediate in glutathione metabolism (18), whereas D-pyroglutamic acid has limited biological activity (19). Therefore, the observed alterations are discussed in the context of L-pyroglutamate as the representative bioactive form. As an intermediate in the glutathione cycle, pyroglutamic acid accumulation is frequently associated with accelerated glutathione turnover and oxidative stress (20). Given that oxidative stress is a recognized contributor to myocardial injury in various cardiovascular conditions (21), the observed increase in pyroglutamic acid may indirectly suggest enhanced oxidative stress in patients who later developed ICI-associated myocarditis, a state that has been associated with inflammatory cytokine and reactive oxygen species release in other myocarditis models (22). This finding complements the report by He et al. (15), who identified elevated L-kynurenine in ICI-associated myocarditis. Although pyroglutamic acid and L-kynurenine are distinct metabolites derived from separate biochemical pathways, glutathione metabolism and the kynurenine pathway, respectively, both converge on the implication of oxidative and immune-related disturbances in ICI-myocarditis. Nevertheless, this case-control design precludes causal inference.

Histamine, an immune-related metabolite primarily released by mast cells and basophils, was decreased in the myocarditis group. Histamine exerts context-dependent effects in inflammatory responses (23). In conventional infectious or autoimmune myocarditis, changes in histamine levels have been inconsistent (24, 25). The observed decrease may reflect altered mast cell activation or enhanced histamine catabolism within the ICI-induced immune microenvironment, though the underlying mechanisms are unknown. Mast cell counts and histamine-metabolizing enzyme expression were not assessed in this study, leaving the biological significance of this finding unresolved. In contrast to histamine, spermine, a polyamine with reported anti-inflammatory and antioxidant properties (26), was increased in the myocarditis group. This elevation is speculated to reflect an attempt to counteract oxidative stress and inflammation, though whether this represents an adaptive or pathological response remains unclear. Notably, altered polyamine metabolism has not been described in prior metabolomic investigations of ICI-associated myocarditis (14, 15), suggesting that spermine may represent a previously unrecognized metabolic feature of this condition.

Apart from the changes in amino acid and polyamine metabolism described above, products related to gut microbiota-host co-metabolism also showed abnormalities. Propionic acid, a short-chain fatty acid with roles in intestinal barrier maintenance and immune regulation (27), was decreased in the myocarditis group, whereas cinnamoylglycine, an aromatic amino acid derivative produced by gut bacterial metabolism (28), was elevated. These reciprocal changes are directly consistent with the established evidence reviewed by Gao et al. (29), who reported that depletion of short-chain fatty acid-producing commensals is associated with increased irAE risk. Although our plasma metabolomics platform cannot identify specific bacterial taxa, the observed decrease in propionic acid and elevation of cinnamoylglycine indirectly suggest that patients who developed ICI-associated myocarditis may harbor gut microbiota dysbiosis characterized by diminished short-chain fatty acid-producing capacity. However, without direct fecal microbiome profiling, the involvement of a “gut-heart axis” remains speculative. Notably, other short-chain fatty acids such as butyrate, acetate, and valerate were not reliably detected in our untargeted metabolomics dataset, likely due to their low physiological concentrations and the technical constraints of the non-targeted platform. Investigating their potential roles in ICI-associated myocarditis using targeted approaches would be an interesting direction for future research.

Unlike the gut microbiota-related metabolites described above, sphingolipid metabolism also showed abnormalities. Ceramide levels tended to decrease in the myocarditis group. This direction of change contrasts with conventional cardiovascular diseases, in which elevated ceramide is typically associated with adverse prognosis (30), suggesting that ICI-associated myocarditis may exhibit a distinct metabolic dysregulation pattern compared with ischemic or infectious etiologies. Within the ICI-treated population, this observation aligns with the consistent theme of lipid metabolic disturbances across ICI studies, although the specific lipid class and direction of change differ: Möhn et al. (13) reported lower triglycerides, Yu et al. (31) identified altered lipid species, whereas our pre-onset cohort showed decreased ceramide, suggesting that lipid metabolic reprogramming in ICI-associated myocarditis may be both class-specific and phase-dependent.

Among the remaining differential metabolites, the biological significance of cyclo-prolylglycine and glycolic acid remains unclear. Phenylpyruvic acid and 1-methylpseudouridine, despite their high loadings on PC1, lack established cardiovascular relevance and may represent dataset-specific signals; their reproducibility should be assessed in independent cohorts. Of note, phenylpyruvic acid showed a markedly lower detection rate in the control group (7/15, 46.7%) compared with the myocarditis group (14/15, 93.3%). This asymmetric distribution explains the bimodal pattern in the violin plot and the large fold-change observed for this metabolite. As phenylpyruvic acid is a gut microbiota-derived metabolite produced by bacterial deamination of phenylalanine, this inter-individual variation likely reflects biological heterogeneity in gut microbial composition rather than technical artifacts. Nevertheless, targeted quantitative validation in independent cohorts is warranted to confirm these findings.

The differential metabolites identified in this study showed limited overlap with those reported in prior metabolomic investigations of ICI-associated myocarditis. Gao et al. (32) reported increased levels of multiple lipid species in acute-phase patients with ICI-myocarditis using targeted lipidomics, whereas our baseline untargeted profiling detected a trend toward decreased ceramide prior to clinical onset, with divergent lipid classes. This directional and metabolic divergence likely reflects distinct metabolic dynamics across disease phases, that is acute injury versus pre-clinical risk. Furthermore, He et al. (15) reported elevated L-kynurenine in ICI-myocarditis, implicating oxidative stress and immune regulation via the JAK/STAT pathway; this study similarly identified elevated pyroglutamic acid, suggesting that metabolic disturbances involving redox imbalance may represent a shared feature of ICI-myocarditis. Nevertheless, L-kynurenine and pyroglutamic acid belong to distinct metabolic pathways. Despite divergent specific metabolites, lipid metabolic disturbances and oxidative stress-related alterations emerge as recurrent themes in ICI-associated myocarditis. Baseline observations in this study extend the metabolic timeline to the pre-clinical window, a temporal dimension not captured in prior acute-phase studies. However, given the paucity of metabolomic studies specifically targeting this condition, cross-study comparability remains limited.

The metabolite PC1 model suggested apparent discriminative ability in this dataset (AUC = 0.964), and was higher than that of the clinical-only model (AUC = 0.618), with marginal further improvement when variables were combined (AUC = 0.973). Decision curve analysis indicated higher net benefit for the combined model within the 20%-40% threshold range. These observations raise the possibility that metabolomic information could contribute to risk identification of ICI-associated myocarditis, pending validation. The modest AUC increment from combining clinical variables (0.973 *vs.* 0.964) further suggests limited incremental contribution from conventional clinical parameters-an observation requiring confirmation in larger cohorts.

This study has several limitations. First, the small sample size (n = 30) limits statistical power and, combined with an Event-Per-Variable (EPV) ratio of 3, renders the models prone to overfitting and optimistic bias (33). The same concern applies to PC1: derived from metabolites preselected for group differences, its AUC of 0.964 largely reflects the selection step rather than true generalizability, and cross-validation does not fully correct this as feature selection was not embedded within the CV loop. Therefore, all performance metrics should be interpreted as exploratory, and the nomogram as a proof-of-concept tool rather than a clinically validated instrument. Second, the lack of an independent validation cohort, the retrospective single-center design, and the inability to map some metabolites to KEGG pathways all constrain the robustness and generalizability of our findings. Third, myoglobin was excluded from the nomogram due to the absence of baseline variability in this matched cohort, limiting the applicability of the clinical model to broader populations. Future prospective, multicenter studies with larger sample sizes and independent validation sets are required to confirm our findings, while orthogonal validation using targeted analytical platforms (e.g., multiple reaction monitoring) and mechanistic studies in cellular and animal models are also warranted.

## 5 Conclusion

In summary, this exploratory study performed untargeted metabolomic profiling on baseline plasma samples from 15 patients with ICI-associated myocarditis and 15 matched controls, delineating a preliminary metabolic landscape associated with subsequent disease development. A total of ten differential metabolites were identified, implicating oxidative stress, immune modulation, and gut microbiota-host interactions in the pre-clinical phase. The metabolite-based model suggested preliminary discriminative ability in this dataset, yet the findings remain strictly hypothesis-generating and warrant prospective validation in independent, large-scale cohorts before any clinical utility can be contemplated.

## Data Availability

The datasets presented in this study can be found in online repositories. The names of the repository/repositories and accession number(s) can be found below: The raw mass spectrometry data generated in this study have been deposited in the MetaboLights repository (https://www.ebi.ac.uk/metabolights/) under the accession number MTBLS15390. The dataset will be publicly available after a 1−year embargo period following publication. Processed metabolite tables and other supporting data are available from the corresponding author upon reasonable request.

## Supplementary material

SUPPLEMENTARY TABLE 1 Detection rates of the ten differential metabolites in the myocarditis and control groups.

Supplementary Figure 1 Principal component analysis (PCA) score plots of quality control (QC) and study samples in positive (A) and negative (B) ion modes. PCA was performed on all filtered metabolites after log2 transformation and Pareto scaling. The tight clustering of QC samples indicates good analytical reproducibility. Blue circles, study samples; red triangles, QC samples. The dashed ellipses represent the 95% confidence regions.

Supplementary Figure 2 Unsupervised PCA score plots of all filtered metabolites in positive (A) and negative (B) ion modes. PCA was performed on all filtered features (242 in positive mode, 148 in negative mode) after log2 transformation and Pareto scaling, without prior knowledge of group labels. (A) Positive ion mode with PC1 (16.3%) and PC2 (14.3%). (B) Negative ion mode with PC1 (15.1%) and PC2 (12.9%). Blue circles, control group; red circles, myocarditis group. Dashed ellipses represent 95% confidence regions. This plot allows readers to assess overall metabolic separation between groups prior to any supervised modeling.

Supplementary Figure 3 Permutation tests (200 permutations) for OPLS-DA models in positive (A) and negative (B) ion modes. Blue dots, R2Y values; red dots, Q2Y values. The dashed lines indicate the intercepts of the regression lines. All permuted Q2Y values were lower than the original model Q2 values (p = 0.005), suggesting that the observed separation was unlikely to be driven by random chance.

Supplementary Figure 4 PCA score plots of the differential metabolites in positive (A) and negative (B) ion modes. PCA was performed on metabolites that met the differential selection criteria (VIP > 1, |log2FC| > 1, q < 0.05). (A) Positive ion mode with PC1 (57.2%) and PC2 (16.3%). (B) Negative ion mode with PC1 (67.7%) and PC2 (14.5%). Blue circles, control group; red circles, myocarditis group. The dashed ellipses represent the 95% confidence regions.

Supplementary Figure 5 Loading plots of differential metabolites on PC1 in positive (A) and negative (B) ion modes. Blue bars indicate metabolites negatively associated with PC1 (higher in control group); red bars indicate metabolites positively associated with PC1 (higher in myocarditis group). Metabolites are ranked by absolute loading values. See Table 2 for the correspondence between PubChem IDs and metabolite names.

Supplementary Figure 6 Calibration curve of the combined logistic regression model for predicting ICI-associated myocarditis. The x-axis represents predicted probability and the y-axis represents observed probability. The dashed diagonal line indicates perfect calibration. Black dots represent mean observed probabilities within each quintile of predicted probabilities, with vertical bars indicating 95% confidence intervals (bootstrap with 1000 resamples). The gray shaded area represents the bootstrap confidence band. Numbers indicate sample size per quintile (n = 6).

Supplementary Figure 7 Decision curve analysis (DCA) comparing the net benefit of the clinical model (blue), metabolite PC1 model (green dashed), and combined model (red) across threshold probabilities for predicting ICI-associated myocarditis. Net benefit is calculated as (true positives / n) - (false positives / n) × (threshold / (1 - threshold)). The gray dashed line represents the “treat all” strategy, and the black line represents the “treat none” strategy. The combined model showed slightly higher net benefit within the 20%-40% threshold range.
