# Supplementary material for: Discovery of plasma metabolomic biomarkers and a predictive model for immune checkpoint inhibitor-associated myocarditis: a pilot case-control study
Source: Front Immunol. 2026 Sep 15;17:1915039. doi: 10.3389/fimmu.2026.1915039 (PMC13619531; doi:10.3389/fimmu.2026.1915039)
Supplement: Supplementary file 1 [file Table1.docx]

Supplementary Table S1. Detection rates of the ten differential metabolites in the myocarditis and control groups

| **Metabolite** | **PubChem ID** | **Ion mode** | **Myocarditis (n=15)** | **Control (n=15)** |
| --- | --- | --- | --- | --- |
| 1-Methylpseudouridine | 99543 | Positive | 15/15 (100%) | 15/15 (100%) |
| Cinnamoylglycine | 709625 | Positive | 15/15 (100%) | 10/15 (66.7%) |
| Ceramide | 5283564 | Positive | 13/15 (86.7%) | 15/15 (100%) |
| Cyclo-Prolylglycine | 126154 | Positive | 15/15 (100%) | 15/15 (100%) |
| Spermine | 1103 | Positive | 15/15 (100%) | 15/15 (100%) |
| Phenylpyruvic acid | 997 | Negative | 14/15 (93.3%) | 7/15 (46.7%) |
| Pyroglutamic acid | 7405 | Positive | 15/15 (100%) | 15/15 (100%) |
| Propionic Acid | 1032 | Negative | 15/15 (100%) | 15/15 (100%) |
| Glycolic Acid | 757 | Negative | 15/15 (100%) | 15/15 (100%) |
| Histamine | 774 | Negative | 15/15 (100%) | 15/15 (100%) |

Notes: Metabolites detected in both ion modes are shown separately per mode. Detection rate defined as the proportion of samples with peak area above the detection limit (log_2_-transformed value > 0).
